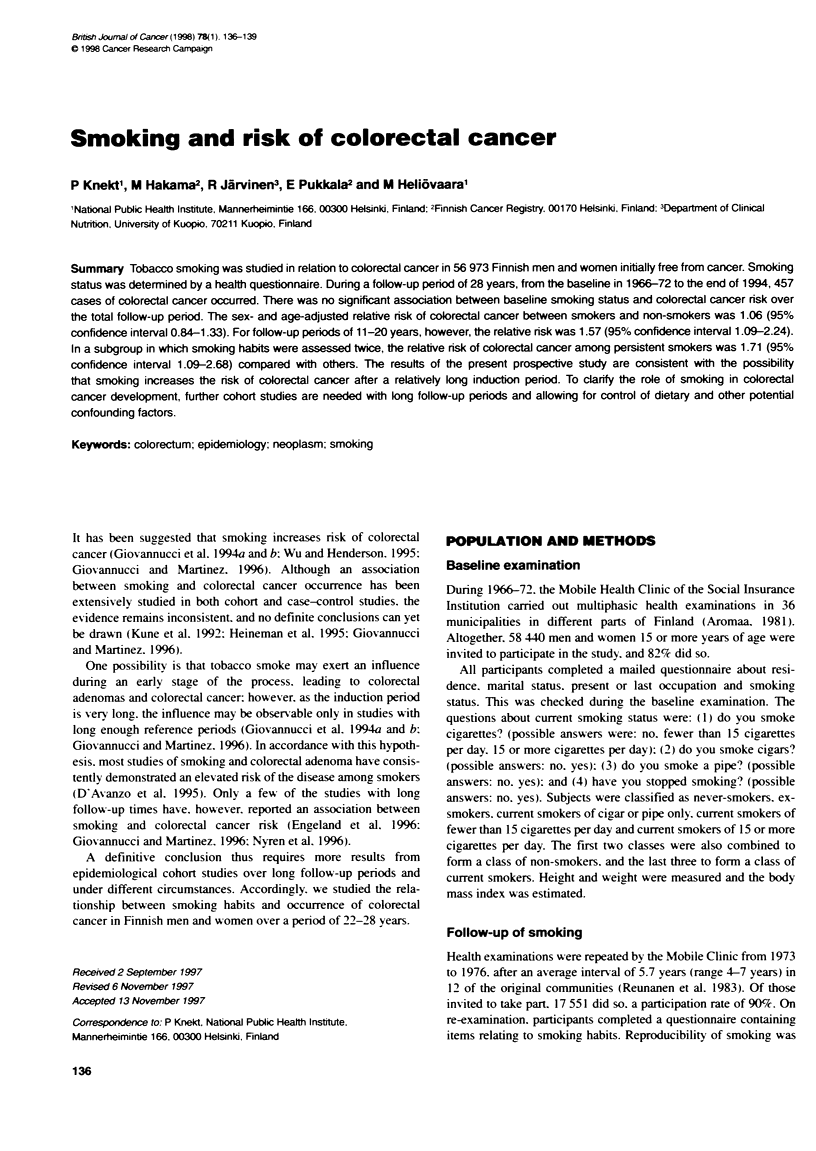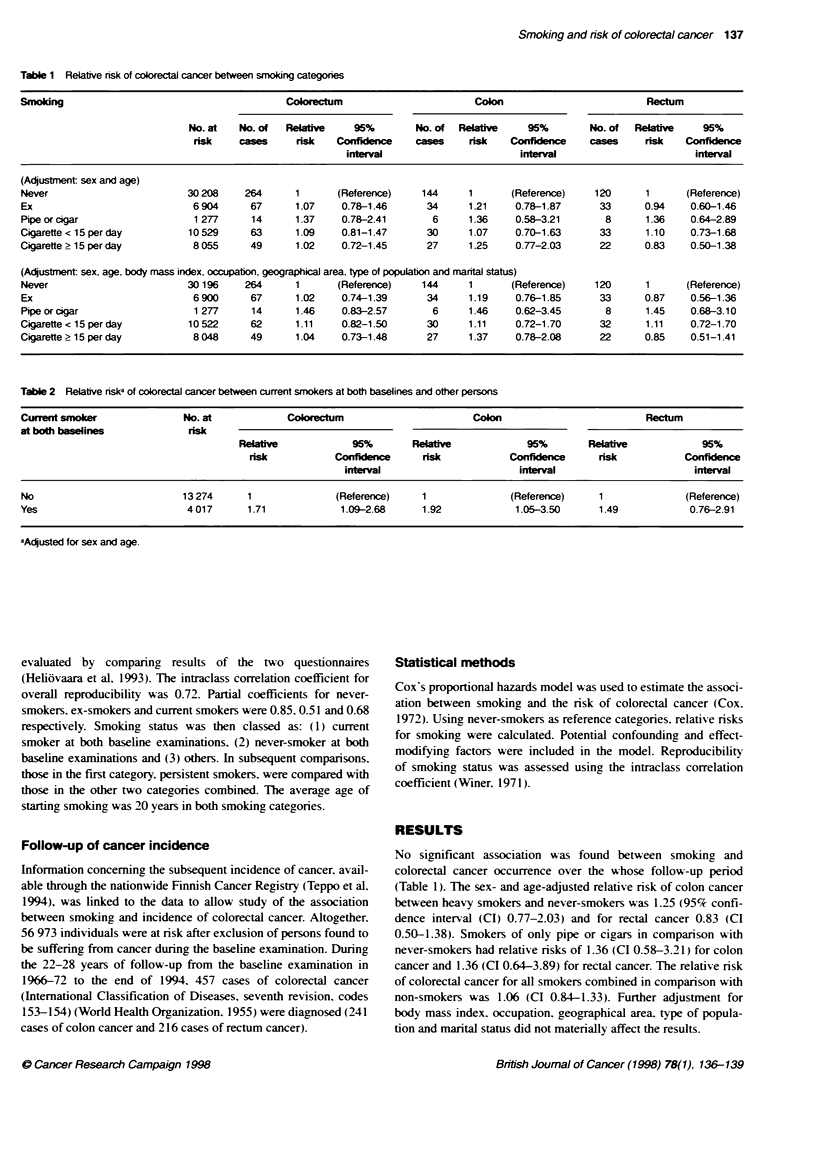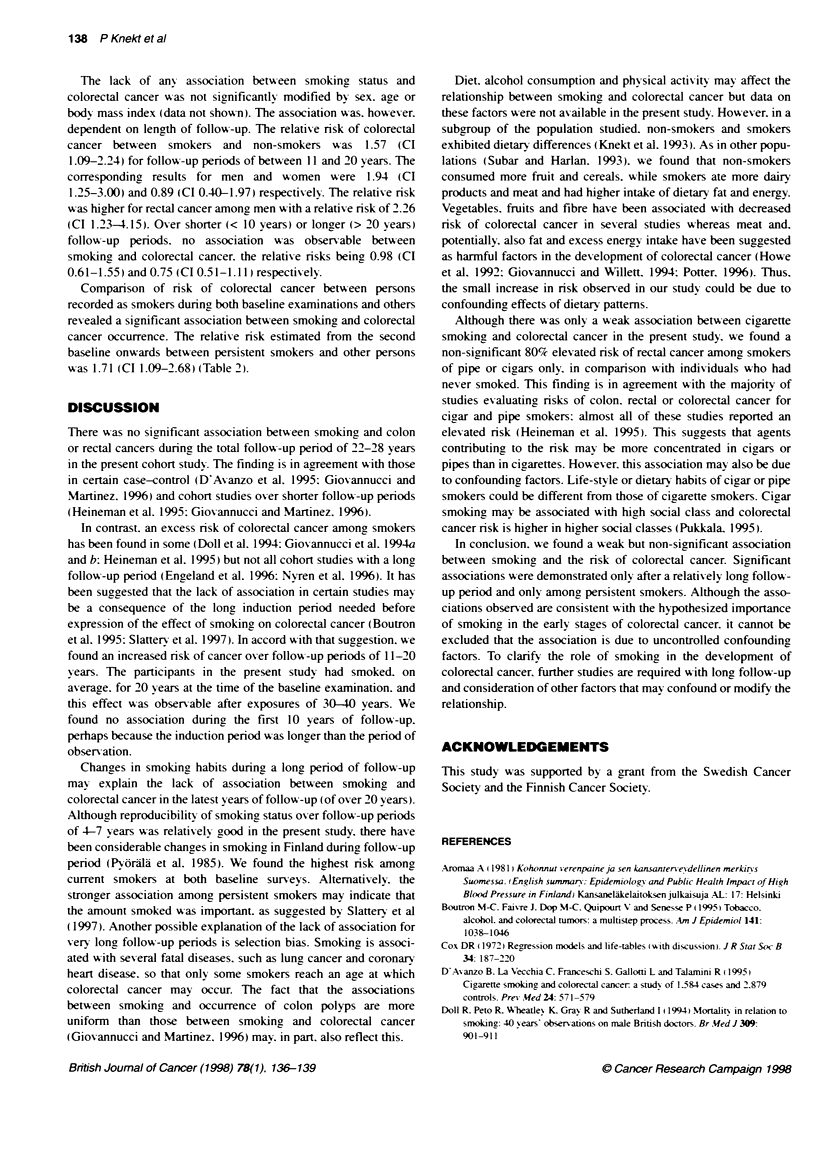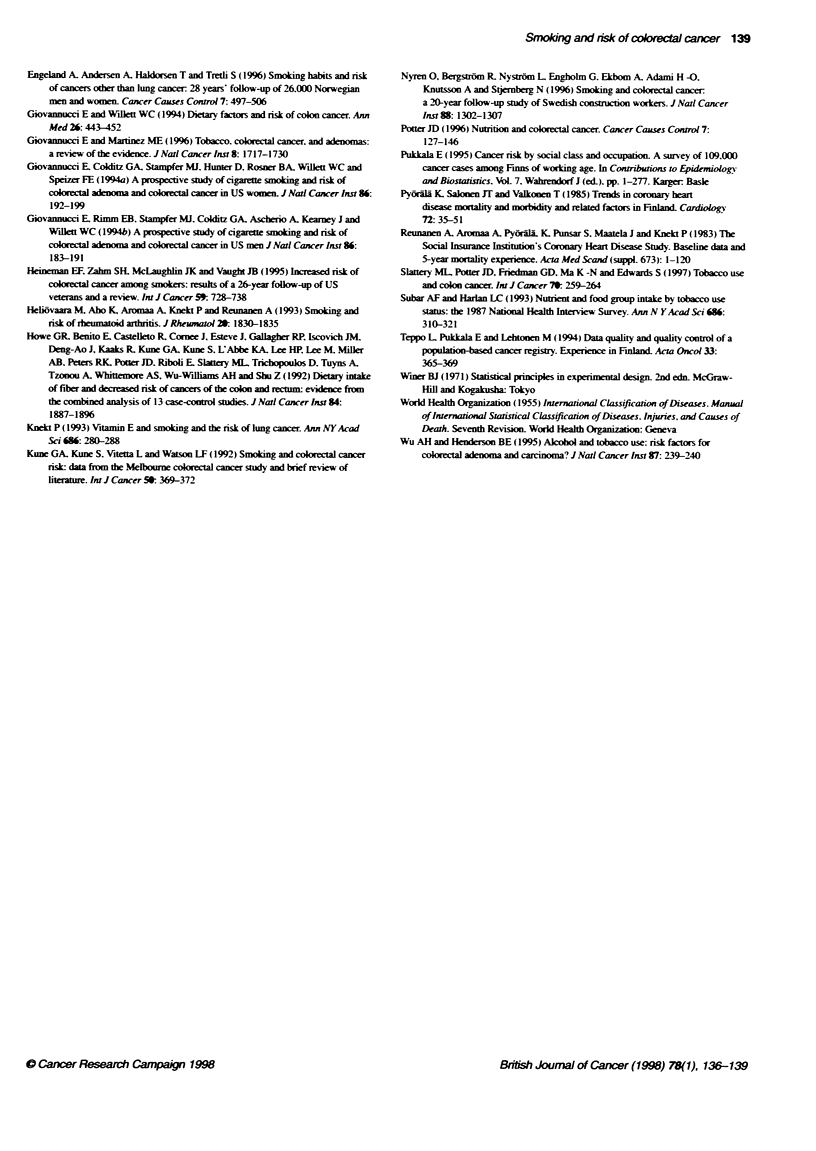# Smoking and risk of colorectal cancer.

**DOI:** 10.1038/bjc.1998.455

**Published:** 1998-07

**Authors:** P. Knekt, M. Hakama, R. Järvinen, E. Pukkala, M. Heliövaara

**Affiliations:** National Public Health Institute, Helsinki, Finland.

## Abstract

Tobacco smoking was studied in relation to colorectal cancer in 56 973 Finnish men and women initially free from cancer. Smoking status was determined by a health questionnaire. During a follow-up period of 28 years, from the baseline in 1966-72 to the end of 1994, 457 cases of colorectal cancer occurred. There was no significant association between baseline smoking status and colorectal cancer risk over the total follow-up period. The sex- and age-adjusted relative risk of colorectal cancer between smokers and non-smokers was 1.06 (95% confidence interval 0.84-1.33). For follow-up periods of 11-20 years, however, the relative risk was 1.57 (95% confidence interval 1.09-2.24). In a subgroup in which smoking habits were assessed twice, the relative risk of colorectal cancer among persistent smokers was 1.71 (95% confidence interval 1.09-2.68) compared with others. The results of the present prospective study are consistent with the possibility that smoking increases the risk of colorectal cancer after a relatively long induction period. To clarify the role of smoking in colorectal cancer development, further cohort studies are needed with long follow-up periods and allowing for control of dietary and other potential confounding factors.


British Journal of Cancer (1998) 78(1), 136–139
© 1998 Cancer Research Campaign

### Smoking and risk of colorectal cancer

**P Knekt[1], M Hakama[2], R Järvinen[3], E Pukkala[2] and M Heliövaara[1]**

[1]National Public Health Institute, Mannerheimintie 166, 00300 Helsinki, Finland; [2]Finnish Cancer Registry, 00170 Helsinki, Finland; [3]Department of Clinical Nutrition, University of Kuopio, 70211 Kuopio, Finland

**Summary** Tobacco smoking was studied in relation to colorectal cancer in 56 973 Finnish men and women initially free from cancer. Smoking status was determined by a health questionnaire. During a follow-up period of 28 years, from the baseline in 1966–72 to the end of 1994, 457 cases of colorectal cancer occurred. There was no significant association between baseline smoking status and colorectal cancer risk over the total follow-up period. The sex- and age-adjusted relative risk of colorectal cancer between smokers and non-smokers was 1.06 (95% confidence interval 0.84–1.33). For follow-up periods of 11–20 years, however, the relative risk was 1.57 (95% confidence interval 1.09–2.24). In a subgroup in which smoking habits were assessed twice, the relative risk of colorectal cancer among persistent smokers was 1.71 (95% confidence interval 1.09–2.68) compared with others. The results of the present prospective study are consistent with the possibility that smoking increases the risk of colorectal cancer after a relatively long induction period. To clarify the role of smoking in colorectal cancer development, further cohort studies are needed with long follow-up periods and allowing for control of dietary and other potential confounding factors.

**Keywords:** colorectum; epidemiology; neoplasm; smoking

It has been suggested that smoking increases risk of colorectal cancer (Giovannucci et al, 1994*a* and *b*; Wu and Henderson, 1995; Giovannucci and Martinez, 1996). Although an association between smoking and colorectal cancer occurrence has been extensively studied in both cohort and case–control studies, the evidence remains inconsistent, and no definite conclusions can yet be drawn (Kune et al, 1992; Heineman et al, 1995; Giovannucci and Martinez, 1996).

One possibility is that tobacco smoke may exert an influence during an early stage of the process, leading to colorectal adenomas and colorectal cancer; however, as the induction period is very long, the influence may be observable only in studies with long enough reference periods (Giovannucci et al, 1994*a* and *b*; Giovannucci and Martinez, 1996). In accordance with this hypothesis, most studies of smoking and colorectal adenoma have consistently demonstrated an elevated risk of the disease among smokers (D'Avanzo et al, 1995). Only a few of the studies with long follow-up times have, however, reported an association between smoking and colorectal cancer risk (Engeland et al, 1996; Giovannucci and Martinez, 1996; Nyren et al, 1996).

A definitive conclusion thus requires more results from epidemiological cohort studies over long follow-up periods and under different circumstances. Accordingly, we studied the relationship between smoking habits and occurrence of colorectal cancer in Finnish men and women over a period of 22–28 years.

*Received 2 September 1997*
*Revised 6 November 1997*
*Accepted 13 November 1997*

*Correspondence to:* P Knekt, National Public Health Institute, Mannerheimintie 166, 00300 Helsinki, Finland

### POPULATION AND METHODS

### Baseline examination

During 1966–72, the Mobile Health Clinic of the Social Insurance Institution carried out multiphasic health examinations in 36 municipalities in different parts of Finland (Aromaa, 1981). Altogether, 58 440 men and women 15 or more years of age were invited to participate in the study, and 82% did so.

All participants completed a mailed questionnaire about residence, marital status, present or last occupation and smoking status. This was checked during the baseline examination. The questions about current smoking status were: (1) do you smoke cigarettes? (possible answers were: no, fewer than 15 cigarettes per day, 15 or more cigarettes per day); (2) do you smoke cigars? (possible answers: no, yes); (3) do you smoke a pipe? (possible answers: no, yes); and (4) have you stopped smoking? (possible answers: no, yes). Subjects were classified as never-smokers, ex-smokers, current smokers of cigar or pipe only, current smokers of fewer than 15 cigarettes per day and current smokers of 15 or more cigarettes per day. The first two classes were also combined to form a class of non-smokers, and the last three to form a class of current smokers. Height and weight were measured and the body mass index was estimated.

### Follow-up of smoking

Health examinations were repeated by the Mobile Clinic from 1973 to 1976, after an average interval of 5.7 years (range 4–7 years) in 12 of the original communities (Reunanen et al, 1983). Of those invited to take part, 17 551 did so, a participation rate of 90%. On re-examination, participants completed a questionnaire containing items relating to smoking habits. Reproducibility of smoking was

**Table 1** Relative risk of colorectal cancer between smoking categories

| Smoking | No. at risk | Colorectum: No. of cases | Colorectum: Relative risk | Colorectum: 95% Confidence interval | Colon: No. of cases | Colon: Relative risk | Colon: 95% Confidence interval | Rectum: No. of cases | Rectum: Relative risk | Rectum: 95% Confidence interval |
|---|---|---|---|---|---|---|---|---|---|---|
| (Adjustment: sex and age) | | | | | | | | | | |
| Never | 30 208 | 264 | 1 | (Reference) | 144 | 1 | (Reference) | 120 | 1 | (Reference) |
| Ex | 6 904 | 67 | 1.07 | 0.78–1.46 | 34 | 1.21 | 0.78–1.87 | 33 | 0.94 | 0.60–1.46 |
| Pipe or cigar | 1 277 | 14 | 1.37 | 0.78–2.41 | 6 | 1.36 | 0.58–3.21 | 8 | 1.36 | 0.64–2.89 |
| Cigarette < 15 per day | 10 529 | 63 | 1.09 | 0.81–1.47 | 30 | 1.07 | 0.70–1.63 | 33 | 1.10 | 0.73–1.68 |
| Cigarette ≥ 15 per day | 8 055 | 49 | 1.02 | 0.72–1.45 | 27 | 1.25 | 0.77–2.03 | 22 | 0.83 | 0.50–1.38 |
| (Adjustment: sex, age, body mass index, occupation, geographical area, type of population and marital status) | | | | | | | | | | |
| Never | 30 196 | 264 | 1 | (Reference) | 144 | 1 | (Reference) | 120 | 1 | (Reference) |
| Ex | 6 900 | 67 | 1.02 | 0.74–1.39 | 34 | 1.19 | 0.76–1.85 | 33 | 0.87 | 0.56–1.36 |
| Pipe or cigar | 1 277 | 14 | 1.46 | 0.83–2.57 | 6 | 1.46 | 0.62–3.45 | 8 | 1.45 | 0.68–3.10 |
| Cigarette < 15 per day | 10 522 | 62 | 1.11 | 0.82–1.50 | 30 | 1.11 | 0.72–1.70 | 32 | 1.11 | 0.72–1.70 |
| Cigarette ≥ 15 per day | 8 048 | 49 | 1.04 | 0.73–1.48 | 27 | 1.37 | 0.78–2.08 | 22 | 0.85 | 0.51–1.41 |

**Table 2** Relative risk[a] of colorectal cancer between current smokers at both baselines and other persons

| Current smoker at both baselines | No. at risk | Colorectum: Relative risk | Colorectum: 95% Confidence interval | Colon: Relative risk | Colon: 95% Confidence interval | Rectum: Relative risk | Rectum: 95% Confidence interval |
|---|---|---|---|---|---|---|---|
| No | 13 274 | 1 | (Reference) | 1 | (Reference) | 1 | (Reference) |
| Yes | 4 017 | 1.71 | 1.09–2.68 | 1.92 | 1.05–3.50 | 1.49 | 0.76–2.91 |

[a]Adjusted for sex and age.

evaluated by comparing results of the two questionnaires (Heliövaara et al, 1993). The intraclass correlation coefficient for overall reproducibility was 0.72. Partial coefficients for never-smokers, ex-smokers and current smokers were 0.85, 0.51 and 0.68 respectively. Smoking status was then classed as: (1) current smoker at both baseline examinations, (2) never-smoker at both baseline examinations and (3) others. In subsequent comparisons, those in the first category, persistent smokers, were compared with those in the other two categories combined. The average age of starting smoking was 20 years in both smoking categories.

### Follow-up of cancer incidence

Information concerning the subsequent incidence of cancer, available through the nationwide Finnish Cancer Registry (Teppo et al, 1994), was linked to the data to allow study of the association between smoking and incidence of colorectal cancer. Altogether, 56 973 individuals were at risk after exclusion of persons found to be suffering from cancer during the baseline examination. During the 22–28 years of follow-up from the baseline examination in 1966–72 to the end of 1994, 457 cases of colorectal cancer (International Classification of Diseases, seventh revision, codes 153–154) (World Health Organization, 1955) were diagnosed (241 cases of colon cancer and 216 cases of rectum cancer).

### Statistical methods

Cox's proportional hazards model was used to estimate the association between smoking and the risk of colorectal cancer (Cox, 1972). Using never-smokers as reference categories, relative risks for smoking were calculated. Potential confounding and effect-modifying factors were included in the model. Reproducibility of smoking status was assessed using the intraclass correlation coefficient (Winer, 1971).

### RESULTS

No significant association was found between smoking and colorectal cancer occurrence over the whose follow-up period (Table 1). The sex- and age-adjusted relative risk of colon cancer between heavy smokers and never-smokers was 1.25 (95% confidence interval (CI) 0.77–2.03) and for rectal cancer 0.83 (CI 0.50–1.38). Smokers of only pipe or cigars in comparison with never-smokers had relative risks of 1.36 (CI 0.58–3.21) for colon cancer and 1.36 (CI 0.64–3.89) for rectal cancer. The relative risk of colorectal cancer for all smokers combined in comparison with non-smokers was 1.06 (CI 0.84–1.33). Further adjustment for body mass index, occupation, geographical area, type of population and marital status did not materially affect the results.

© Cancer Research Campaign 1998

The lack of any association between smoking status and colorectal cancer was not significantly modified by sex, age or body mass index (data not shown). The association was, however, dependent on length of follow-up. The relative risk of colorectal cancer between smokers and non-smokers was 1.57 (CI 1.09–2.24) for follow-up periods of between 11 and 20 years. The corresponding results for men and women were 1.94 (CI 1.25–3.00) and 0.89 (CI 0.40–1.97) respectively. The relative risk was higher for rectal cancer among men with a relative risk of 2.26 (CI 1.23–4.15). Over shorter (< 10 years) or longer (> 20 years) follow-up periods, no association was observable between smoking and colorectal cancer, the relative risks being 0.98 (CI 0.61–1.55) and 0.75 (CI 0.51–1.11) respectively.

Comparison of risk of colorectal cancer between persons recorded as smokers during both baseline examinations and others revealed a significant association between smoking and colorectal cancer occurrence. The relative risk estimated from the second baseline onwards between persistent smokers and other persons was 1.71 (CI 1.09–2.68) (Table 2).

### DISCUSSION

There was no significant association between smoking and colon or rectal cancers during the total follow-up period of 22–28 years in the present cohort study. The finding is in agreement with those in certain case–control (D'Avanzo et al, 1995; Giovannucci and Martinez, 1996) and cohort studies over shorter follow-up periods (Heineman et al, 1995; Giovannucci and Martinez, 1996).

In contrast, an excess risk of colorectal cancer among smokers has been found in some (Doll et al, 1994; Giovannucci et al, 1994*a* and *b*; Heineman et al, 1995) but not all cohort studies with a long follow-up period (Engeland et al, 1996; Nyren et al, 1996). It has been suggested that the lack of association in certain studies may be a consequence of the long induction period needed before expression of the effect of smoking on colorectal cancer (Boutron et al, 1995; Slattery et al, 1997). In accord with that suggestion, we found an increased risk of cancer over follow-up periods of 11–20 years. The participants in the present study had smoked, on average, for 20 years at the time of the baseline examination, and this effect was observable after exposures of 30–40 years. We found no association during the first 10 years of follow-up, perhaps because the induction period was longer than the period of observation.

Changes in smoking habits during a long period of follow-up may explain the lack of association between smoking and colorectal cancer in the latest years of follow-up (of over 20 years). Although reproducibility of smoking status over follow-up periods of 4–7 years was relatively good in the present study, there have been considerable changes in smoking in Finland during follow-up period (Pyörälä et al, 1985). We found the highest risk among current smokers at both baseline surveys. Alternatively, the stronger association among persistent smokers may indicate that the amount smoked was important, as suggested by Slattery et al (1997). Another possible explanation of the lack of association for very long follow-up periods is selection bias. Smoking is associated with several fatal diseases, such as lung cancer and coronary heart disease, so that only some smokers reach an age at which colorectal cancer may occur. The fact that the associations between smoking and occurrence of colon polyps are more uniform than those between smoking and colorectal cancer (Giovannucci and Martinez, 1996) may, in part, also reflect this.

Diet, alcohol consumption and physical activity may affect the relationship between smoking and colorectal cancer but data on these factors were not available in the present study. However, in a subgroup of the population studied, non-smokers and smokers exhibited dietary differences (Knekt et al, 1993). As in other populations (Subar and Harlan, 1993), we found that non-smokers consumed more fruit and cereals, while smokers ate more dairy products and meat and had higher intake of dietary fat and energy. Vegetables, fruits and fibre have been associated with decreased risk of colorectal cancer in several studies whereas meat and, potentially, also fat and excess energy intake have been suggested as harmful factors in the development of colorectal cancer (Howe et al, 1992; Giovannucci and Willett, 1994; Potter, 1996). Thus, the small increase in risk observed in our study could be due to confounding effects of dietary patterns.

Although there was only a weak association between cigarette smoking and colorectal cancer in the present study, we found a non-significant 80% elevated risk of rectal cancer among smokers of pipe or cigars only, in comparison with individuals who had never smoked. This finding is in agreement with the majority of studies evaluating risks of colon, rectal or colorectal cancer for cigar and pipe smokers; almost all of these studies reported an elevated risk (Heineman et al, 1995). This suggests that agents contributing to the risk may be more concentrated in cigars or pipes than in cigarettes. However, this association may also be due to confounding factors. Life-style or dietary habits of cigar or pipe smokers could be different from those of cigarette smokers. Cigar smoking may be associated with high social class and colorectal cancer risk is higher in higher social classes (Pukkala, 1995).

In conclusion, we found a weak but non-significant association between smoking and the risk of colorectal cancer. Significant associations were demonstrated only after a relatively long follow-up period and only among persistent smokers. Although the associations observed are consistent with the hypothesized importance of smoking in the early stages of colorectal cancer, it cannot be excluded that the association is due to uncontrolled confounding factors. To clarify the role of smoking in the development of colorectal cancer, further studies are required with long follow-up and consideration of other factors that may confound or modify the relationship.

### ACKNOWLEDGEMENTS

This study was supported by a grant from the Swedish Cancer Society and the Finnish Cancer Society.

### REFERENCES

Aromaa A (1981) *Kohonnut verenpaine ja sen kansanterveydellinen merkitys Suomessa. (English summary: Epidemiology and Public Health Impact of High Blood Pressure in Finland)* Kansaneläkelaitoksen julkaisuja AL: 17: Helsinki

Boutron M-C, Faivre J, Dop M-C, Quipourt V and Senesse P (1995) Tobacco, alcohol, and colorectal tumors: a multistep process. *Am J Epidemiol* **141**: 1038–1046

Cox DR (1972) Regression models and life-tables (with discussion). *J R Stat Soc B* **34**: 187–220

D'Avanzo B, La Vecchia C, Franceschi S, Gallotti L and Talamini R (1995) Cigarette smoking and colorectal cancer: a study of 1,584 cases and 2,879 controls. *Prev Med* **24**: 571–579

Doll R, Peto R, Wheatley K, Gray R and Sutherland I (1994) Mortality in relation to smoking: 40 years' observations on male British doctors. *Br Med J* **309**: 901–911

© Cancer Research Campaign 1998

Engeland A, Andersen A, Haldorsen T and Tretli S (1996) Smoking habits and risk of cancers other than lung cancer: 28 years' follow-up of 26.000 Norwegian men and women. *Cancer Causes Control* **7**: 497–506

Giovannucci E and Willett WC (1994) Dietary factors and risk of colon cancer. *Ann Med* **26**: 443–452

Giovannucci E and Martinez ME (1996) Tobacco, colorectal cancer, and adenomas: a review of the evidence. *J Natl Cancer Inst* **8**: 1717–1730

Giovannucci E, Colditz GA, Stampfer MJ, Hunter D, Rosner BA, Willett WC and Speizer FE (1994*a*) A prospective study of cigarette smoking and risk of colorectal adenoma and colorectal cancer in US women. *J Natl Cancer Inst* **86**: 192–199

Giovannucci E, Rimm EB, Stampfer MJ, Colditz GA, Ascherio A, Kearney J and Willett WC (1994*b*) A prospective study of cigarette smoking and risk of colorectal adenoma and colorectal cancer in US men *J Natl Cancer Inst* **86**: 183–191

Heineman EF, Zahm SH, McLaughlin JK and Vaught JB (1995) Increased risk of colorectal cancer among smokers: results of a 26-year follow-up of US veterans and a review. *Int J Cancer* **59**: 728–738

Heliövaara M, Aho K, Aromaa A, Knekt P and Reunanen A (1993) Smoking and risk of rheumatoid arthritis. *J Rheumatol* **20**: 1830–1835

Howe GR, Benito E, Castelleto R, Cornee J, Esteve J, Gallagher RP, Iscovich JM, Deng-Ao J, Kaaks R, Kune GA, Kune S, L'Abbe KA, Lee HP, Lee M, Miller AB, Peters RK, Potter JD, Riboli E, Slattery ML, Trichopoulos D, Tuyns A, Tzonou A, Whittemore AS, Wu-Williams AH and Shu Z (1992) Dietary intake of fiber and decreased risk of cancers of the colon and rectum: evidence from the combined analysis of 13 case-control studies. *J Natl Cancer Inst* **84**: 1887–1896

Knekt P (1993) Vitamin E and smoking and the risk of lung cancer. *Ann NY Acad Sci* **686**: 280–288

Kune GA, Kune S, Vitetta L and Watson LF (1992) Smoking and colorectal cancer risk: data from the Melbourne colorectal cancer study and brief review of literature. *Int J Cancer* **50**: 369–372

Nyren O, Bergström R, Nyström L, Engholm G, Ekbom A, Adami H -O, Knutsson A and Stjernberg N (1996) Smoking and colorectal cancer: a 20-year follow-up study of Swedish construction workers. *J Natl Cancer Inst* **88**: 1302–1307

Potter JD (1996) Nutrition and colorectal cancer. *Cancer Causes Control* **7**: 127–146

Pukkala E (1995) Cancer risk by social class and occupation. A survey of 109.000 cancer cases among Finns of working age. In *Contributions to Epidemiology and Biostatistics*, Vol. 7, Wahrendorf J (ed.), pp. 1–277. Karger: Basle

Pyörälä K, Salonen JT and Valkonen T (1985) Trends in coronary heart disease mortality and morbidity and related factors in Finland. *Cardiology* **72**: 35–51

Reunanen A, Aromaa A, Pyörälä, K, Punsar S, Maatela J and Knekt P (1983) The Social Insurance Institution's Coronary Heart Disease Study. Baseline data and 5-year mortality experience. *Acta Med Scand* (suppl. 673): 1–120

Slattery ML, Potter JD, Friedman GD, Ma K -N and Edwards S (1997) Tobacco use and colon cancer. *Int J Cancer* **70**: 259–264

Subar AF and Harlan LC (1993) Nutrient and food group intake by tobacco use status: the 1987 National Health Interview Survey. *Ann N Y Acad Sci* **686**: 310–321

Teppo L, Pukkala E and Lehtonen M (1994) Data quality and quality control of a population-based cancer registry. Experience in Finland. *Acta Oncol* **33**: 365–369

Winer BJ (1971) Statistical principles in experimental design. 2nd edn. McGraw-Hill and Kogakusha: Tokyo

World Health Organization (1955) *International Classification of Diseases. Manual of International Statistical Classification of Diseases, Injuries, and Causes of Death*. Seventh Revision. World Health Organization: Geneva

Wu AH and Henderson BE (1995) Alcohol and tobacco use: risk factors for colorectal adenoma and carcinoma? *J Natl Cancer Inst* **87**: 239–240

© Cancer Research Campaign 1998